# Identification and characterisation of short chain rhamnolipid production in a previously uninvestigated, non-pathogenic marine pseudomonad

**DOI:** 10.1007/s00253-018-9202-3

**Published:** 2018-07-10

**Authors:** Matthew S. Twigg, L. Tripathi, A. Zompra, K. Salek, V. U. Irorere, T. Gutierrez, G. A. Spyroulias, R. Marchant, I. M. Banat

**Affiliations:** 1 School of Biomedical Sciences, Ulster University, Coleraine, Northern Ireland BT52 1SA UK; 2 Institute of Mechanical, Process and Energy Engineering, School of Engineering and Physical Sciences, Heriot-Watt University, Edinburgh, EH14 4AS UK; 3 Department of Pharmacy, University of Patras, 26504 Patras, Greece

**Keywords:** Biosurfactant, Rhamnolipid, *Pseudomonas*, Marine bacteria, Non-pathogenic

## Abstract

This study aimed to identify and characterise biosurfactant compounds produced by bacteria associated with a marine eukaryotic phytoplankton bloom. One strain, designated MCTG214(3b1), was isolated by enrichment with polycyclic aromatic hydrocarbons and based on 16S rDNA, and *gyrB* sequencing was found to belong to the genus *Pseudomonas*, however not related to *P. aeruginosa*. Cell-free supernatant samples of strain MCTG214(3b1) at stationary phase showed significant reductions in surface tension. HPLC-MS and NMR analysis of these samples indicated the presence of five different rhamnolipid (RL) congeners. Di-rhamnolipids accounted for 87% relative abundance and all congeners possessed fatty acid moieties consisting of 8–12 carbons. PCR screening of strain MCTG214(3b1) DNA revealed homologues to the *P. aeruginosa* RL synthesis genes *rhlA* and *rhlB*; however, no *rhlC* homologue was identified. Using the *Galleria mellonella* larvae model, strain MCTG214(3b1) was demonstrated to be far less pathogenic than *P. aeruginosa*. This study identifies for the first time a significantly high level of synthesis of short chain di-rhamnolipids by a non-pathogenic marine *Pseudomonas* species. We postulate that RL synthesis in *Pseudomonas* sp. MCTG214(3b1) is carried out by enzymes expressed from *rhlA*/*B* homologues similar to those of *P. aeruginosa*; however, a lack of *rhlC* potentially indicates the presence of a second novel rhamnosyltransferase responsible for the di-rhamnolipid congeners identified by HPLC-MS.

**Electronic supplementary material:**

The online version of this article (10.1007/s00253-018-9202-3) contains supplementary material, which is available to authorized users.

## Introduction

Biosurfactants are amphipathic compounds comprising hydrophobic and hydrophilic moieties that confer on them the ability to interface with non-soluble and soluble substances. These compounds have been shown to be synthesised by a wide variety of microbial taxa, function to reduce tension at interfacial surfaces and have many industrial applications (Desai and Banat 1997; Banat et al. 2010). The most ubiquitous group of bacterial synthesised biosurfactants is glycolipids of which the best characterised class is the rhamnolipids (RLs) (Desai and Banat 1997; Abdel-Mawgoud et al. 2010). RLs are composed of either one or two rhamnose monosaccharides covalently bonded with up to two 3-(hydroxyalkanoyloxy)alkanoic acid (HAA) fatty acid tails ranging between 8 and 16 carbons in length (Abdel-Mawgoud et al. 2010). Bacterial RL biosynthesis was first elucidated in the Gram negative, opportunistic pathogen *Pseudomonas aeruginosa* (Bergstrom et al. 1946; Jarvis and Johnson 1949; Rudden et al. 2015). Although *P. aeruginosa* can synthesise a range of RL congeners, di-rhamnolipids are the most abundant, with Rha-Rha-C_10_-C_10_ predominating (Rudden et al. 2015). Mono-rhamnolipids are also produced by *P. aeruginosa* as precursors to di-rhamnolipid; strains only synthesising mono-rhamnolipids are rare (Déziel et al. 1999; Rudden et al. 2015). RL synthesis is achieved using three separate enzymes: RhlA, responsible for the synthesis of the fatty acid dimer precursor moieties (HAA); RhlB, a rhamnosyltransferase enzyme which conjugates HAA to dTDP-L-rhamnose to form mono-rhamnolipid and RhlC, a second rhamnosyltransferase enzyme that utilises mono-rhamnolipid as substrate adding a second dTDP-L-rhamnose to form di-rhamnolipid (Ochsner et al. 1994; Rahim et al. 2001; Deziel et al. 2003). These enzymes are expressed from the genes *rhlA* and *rhlB*, located on a single operon alongside an AHL-mediated quorum sensing system (*rhlRI*) and *rhlC*, which is located approx. 2.5 Mb downstream of the *rhl* operon (Ochsner et al. 1994).

The global production of surfactant compounds for industrial use was estimated at 13 million tonnes per annum (Marchant and Banat 2012), with the majority of these compounds derived from organic chemical synthesis using petrochemicals. Petrochemical-derived surfactant production is detrimental for two important reasons: (i) the use of non-renewable sources for their synthesis and (ii) the toxicological effects these synthetic compounds exert upon humans and the environment (Dreja et al. 2012; Franzetti et al. 2012). Industries are increasingly investigating biosurfactants as replacements for their synthetic counterparts. A major impediment to this is low yield and high production costs associated with biosurfactant production. This highlights the important need to continually pursue alternative microorganisms that are capable of producing commercially exploitable yields of biosurfactants such as RLs (i.e. > 2000 μg ml^−1^ crude extract) (Marchant and Banat 2012).

Although there has been a significant amount of research on RL synthesis in *P. aeruginosa*, large-scale production and exploitation are limited due to the pathogenicity of *P. aeruginosa*. A number of different strategies are currently being investigated that either involve identifying an alternative (non-pathogenic) source of RL for commercial use or to genetically modify a non-pathogenic organism to synthesise RLs. Cabrera-Valladares et al. (2006) reported the expression of *rhlA*, *rhlB* and *rhlC* from *P. aeruginosa* PAO1 in *Escherichia coli* K12; however, RL yield was significantly lower than that in the original donor organism (120 μg ml^−1^ compared with 229 μg ml^−1^) (Cabrera-Valladares et al. 2006). Other studies have attempted similar strategies using “non-pathogenic” *Pseudomonas* species as hosts; although yields were improved compared to recombinant *E. coli* (1500 μg ml^−1^), they were still substantially lower than in wild-type strains of *P. aeruginosa* that when cultured under optimal fermentation conditions can produce yields of up to 40,000 μg ml^−1^ (Wittgens et al. 2011). Reduced yields observed with these approaches may be attributed to the complex level of genetic regulation involved in the synthesis of RL in *P. aeruginosa*, which includes layered quorum sensing systems (Pearson et al. 1997). Recently, Grosso-Becerra et al. (2016) reported recombinant expression of *rhlA* and *rhlB* in a “non-pathogenic” *P. aeruginosa* strain (ATCC9027); this genetic manipulation of the host produced yields comparable to *P. aeruginosa* PAO1 (approx. yields of 120 μg ml^−1^ for both the “non-pathogenic” *P. aeruginosa* and wild-type PAO1). By omitting the *rhlC* gene, they were able to engineer a strain that exclusively synthesises mono-rhamnolipid (Grosso-Becerra et al. 2016). Although this approach appears at first to be highly promising from a biotechnological view, many commercial companies are still reluctant to utilise any *P. aeruginosa* strain due to its bio-safety category of 2.

As an alternative to recombinant synthesis of RLs, a number of studies have identified bacterial species, other than *P. aeruginosa*, that can synthesise RLs. The best-studied example is *Burkholderia thailandensis* which has been shown to synthesis RLs during the late stationary phase of growth (Dubeau et al. 2009; Funston et al. 2016). Interestingly, the RLs synthesised by *B. thailandensis* significantly differ from those synthesised by *P. aeruginosa*. *B. thailandensis* synthesises longer chain RLs with Rha-Rha-C_14_-C_14_ being the most abundant congener compared to RLs with acyl chains possessing 10–12 carbons, predominantly seen in *P. aeruginosa* (Rudden et al. 2015; Funston et al. 2016). Synthesis is achieved via a similar mechanism to that found in *P. aeruginosa*; however, the RL synthesis enzymes are expressed from *rhlA-C* genes that share only 40% sequence identity with those of *P. aeruginosa*, offering an explanation for the differing congener profile (Dubeau et al. 2009; Funston et al. 2016). Recently, RL production in *B. thailandensis* has been linked to polyhydroxyalkanoate (PHA) synthesis and storage. *B. thailandensis* PHA synthase mutants produce significantly higher yields of RL in comparison to the wild-type strain (Funston et al. 2017). It should, however, be noted that the longer chain RL congeners synthesised by *B. thailandensis* possess differing activities to the shorter chain congeners synthesised by *P. aeruginosa*, which could have a marked effect on their potential biotechnological application (Elshikh et al. 2017). In addition to *B. thailandensis*, a number of *Pseudomonas* species, unrelated to *P. aeruginosa*, have been putatively identified as capable of producing RLs. These include *Pseudomonas fluorescens*, *Pseudomonas chlororaphis*, *Pseudomonas putida* and various others for which species identification has not been determined (Gunther et al. 2005; Vasileva-Tonkova et al. 2006; Martinez-Toledo et al. 2006; Vasileva-Tonkova et al. 2011; Nordin et al. 2013). In many of these studies, RL production was not confirmed by robust qualitative analytical techniques. Additionally, the taxonomic identity of many of these strains has been carried out using unreliable culture-dependent techniques (Irorere et al. 2017).

Here, we report the isolation of a hydrocarbon degrading marine bacterium belonging to the genus *Pseudomonas*. Using molecular biology techniques, we show this strain to be taxonomically un-related to *P. aeruginosa* and, using the *Galleria mellonella* (Wax Worm) larvae model, can be putatively classified as non-pathogenic compared to *P. aeruginosa*. In accordance with the criteria listed by Irorene et al. (2017), the strain was subsequently assessed for evidence of surface-active compound production with RL synthesis being confirmed and characterised by both HPLC-MS and nuclear magnetic resonance (NMR) spectroscopy. Finally, we report that this strain possesses homologues to the RL synthesis genes *rhlA* and *rhlB*, hence providing evidence for RL synthesis by a new type of non-pathogenic *Pseudomonas*.

## Materials and methods

### Isolation of strain MCTG214(3b1)

Water samples were collected (0.5 m depth) on 29 July 2009, from Sarasota Bay (station 4) in Florida (27.4132° N, − 82.5777° W) during a bloom of *Protoperidinium*. To isolate polycyclic aromatic hydrocarbon (PAH)-degrading bacteria, enrichment cultures were prepared using acid-washed (0.1 M HCl), steam-sterilised glass test tubes (16 × 125 mm) fitted with screw caps lined with aluminium foil to prevent PAH loss via adsorption. Stock solutions (ca. 3000 mg l^−1^) of phenanthrene, anthracene, fluorene and pyrene dissolved in acetone were prepared. Four sets of six test tubes each were prepared containing one of each of the PAHs and 2.8 ml of ONR7a medium (Dyksterhouse et al. 1995), and then inoculated with 200 μl of the water sample from Sarasota Bay. The test tubes were incubated in the dark with gentle shaking (100 rpm) at 21 °C. After 2 and 4 weeks of incubation, 5-μl samples were taken from each PAH incubation and streaked onto ONR7a agar plates that were then sprayed with the respective PAH compound used in the enrichments (Kiyohara et al. 1982). The plates were then stored in closed plastic bags in the dark at room temperature. Colonies forming clearing zones were picked, purified and stored frozen at − 80 °C with the addition of 20% (*v*/*v*) glycerol (Sigma-Aldrich). Following isolation, one strain, designated MCTG214(3b1), was selected for study based on its ability to produce a biosurfactant agent that significantly reduced the surface tension of water. The strain was routinely cultured at 30 °C in Zobell’s 2216 Marine Broth (ZMB). ZMB was composed of 5 g l^−1^ Bacto-Peptone (*BD Biosciences*) and 1 g l^−1^ yeast extract (Sigma-Aldrich) in artificial sea water (Cold Spring Harbour Laboratory 2012) supplemented with 100× marine supplements (1:100) and 1% (*w*/*v*) glucose (Sigma-Aldrich) (Zobell 1941).

### Optimisation of MCTG214(3b1) growth parameters

The growth of MCTG214(3b1) in response to different physical and medium conditions was assessed in shake flask culture. A 50-ml seed culture of MCTG214(3b1) was grown in ZMB supplemented with 1% (*w*/*v*) glucose for 18 h in a 250-ml Erlenmeyer flask. In 500-ml Erlenmeyer flasks, the seed culture was used to inoculate 100 ml ZMB incubated for 48 h with bacterial growth assessed via measurement of optical density (OD) at 600 nm (*Pharmacia Biotech* Novaspec II) at 2, 4, 6, 12, 24 and 48 h growth. Physical and media conditions tested included incubation temperature (25, 27, 30 and 37 °C); initial media pH with the bacteria incubated at 30 °C in media with a defined pH of 4.0, 5.5, 7.0 and 8.5; media salinity with the bacteria incubated at 30 °C in media with a concentration of 0, 5, 10, 20, 30 and 40 g l^−1^ sea salts (Sigma-Aldrich). Additionally, bacterial growth at 30 °C was assessed with and without the addition of glucose, with and without the addition of marine supplements and in the presence of equal amounts of nitrogen from differing sources (yeast extract/peptone, urea, NaNO_3_ and NH_4_NO_3_). Testing of each physical and/or media condition was carried out using triplicate cultures.

### Determination of viable counts and surface tension reduction

A 50-ml seed culture of MCTG214(3b1) was grown in ZMB supplemented with 1% (*w*/*v*) glucose for 18 h in a 250-ml Erlenmeyer flask. The seed culture was used to inoculate triplicate 1-l Erlenmeyer flasks containing 100 ml of ZMB supplemented with 1% (*v*/*v*) rapeseed oil (Sigma-Aldrich) to an initial OD_600 nm_ of 0.01 and incubated at 30 °C. At 4, 8, 18, 24, 48 and 72 h, 1 ml from each triplicate flask was removed and dilutions (10^−3^–10^−8^) prepared with ZMB. Samples (20 μl) of each dilution were then spotted to ZMB agar plates for bacterial growth assessment via colony forming unit (CFU) determination using the Miles and Misra method. The remaining culture was centrifuged (10,000×*g*; 30 min) to pellet the bacterial cells. The resultant cell-free supernatant was transferred to a sterile glass vessel. The surface tension of cell-free supernatant samples was measured via the Du Noüy ring method using a digital tensiometer (*Krüss* K10ST). For comparison, the surface tension of freshly inoculated culture media (*t* = 0 h) and of distilled water using the same method.

### Extraction and chemical analysis of RL

To gain a RL production profile, 90 ml of ZMB + 1% (*v*/*v*) rapeseed oil was inoculated with 10 ml of a MCTG214(3b1) seed culture that had been incubated at 30 °C in ZMB supplemented with 1% (*w*/*v*) glucose for 18 h. Following incubation for 96 h at 30 °C the culture was centrifuged (13,000×*g*; 20 min) to remove most of the cellular biomass. The resultant supernatant was collected, acidified to pH 2.0 with 1 M HCl (Sigma-Aldrich) and extracted three times with an equal volume of ethyl acetate (Sigma-Aldrich). The organic phase was collected, dried with 0.5 g anhydrous MgSO4 (Sigma-Aldrich) per 100 ml ethyl acetate, then filtered and rotary evaporated to obtain a crude extract containing RLs. All crude extracts were measured gravimetrically. To remove any unwanted impurities from the RL extracts, solid phase extraction (SPE) was carried out using Strata SI-1 Silica (55 μm, 70 Å) Giga tubes (*Phenomenex*), and the extracts again measured gravimetrically prior to further analysis (Marchant and Banat 2014). The determination of the relative proportion of rhamnolipid present in the extracts was carried out after SPE purification by HPLC-MS. The system used consisted of a LCQ classic MATT ion trap mass spectrometer (*ThermoFinnigan*) operated in the negative ionisation mode and coupled to a thermo system LC P4000 (*ThermoFinnigan*) high-performance liquid chromatograph equipped with a 150 × 4.6 mm Kinetex 5 μM F5 100 Å LC column. HPLC-grade water and acetonitrile were used as mobile phases 1 and 2, respectively, while m/z range was 110–1200. The sample injection volume was 10 μl.

1D and 2D NMR analyses were carried out using a Bruker Avance III High-Definition, four-channel 700 MHz NMR spectrometer equipped with a cryogenically cooled 5 mm ^1^H/^13^C/^15^N/^2^H Z-gradient probe. All analyses were carried out at 298 K in the solvent system CDCl_3_/MeOD (70:30, *v*/*v*). ^1^H and ^13^C chemical shifts are reported in parts per million (ppm) relative to residual CDCl_3_ signal at 7.26 ppm for protons and 77.16 ppm for carbons, respectively. Correlation spectroscopy (COSY), heteronuclear single quantum coherence (HSQC) and heteronuclear multiple-bond correlation spectroscopy (HMBC) were all performed. Assignment of the spectra was carried out according to the literature for glycolipids and rhamnolipids (Bubb 2003; Jensen et al. 2007; Kügler et al. 2014; Chen et al. 2016; Tedesco et al. 2016).

### DNA extraction, amplification and sequencing

Chromosomal DNA was extracted from approx. 1 × 10^9^ cells of MCTG214(3b1) using a DNeasy Blood and Tissue Kit (*Qiagen*) as per the manufacturer’s instructions. DNA was amplified by the polymerase chain reaction (PCR) using primers specific for each target gene (Table S1). PCR amplification was carried out in a *Techne* TC-5000 thermocycler using recombinant *Taq* DNA Polymerase (*Thermo Fisher Scientific*). Reactions were made as per the manufacturer’s instructions in a total volume of 50 μl using 50 ng template DNA. An initial denaturation step of 94 °C for 3 min followed by 30 cycles of 94 °C for 45 s, specific primer annealing temperature (Table S1) for 30 s and elongation at 72 °C for 90 s/kb of target DNA. PCR products were separated based on molecular weight using a 1% (*w*/*v*) agarose gel made with TBE buffer containing a final concentration of 100 mM Tris, 90 mM boric acid and 1 mM EDTA (*Thermo Fisher Scientific*). DNA was visualised under UV light using SybrSafe DNA stain (*Thermo Fisher Scientific*). Amplified DNA was purified using a Wizard Gel and PCR Clean-up System (*Promega*) and then sequenced using the Sanger sequencing technique by *GATC Biotech* (Cologne, Germany) with primers specific for each target gene (Table S1).

### Cloning of RL synthesis genes

Purified RL biosynthesis gene DNA was ligated into pGEM T-Easy (*Promega*) in a 10-μl reaction using a 1:3 ratio of vector DNA to insert DNA, T4 DNA Ligase (*Promega*) and rapid DNA ligase buffer (*Promega*). DNA ligation reactions were carried at 22 °C for 60 min (Sambrook and Russell 2001). Resultant pGEM vector products containing inserted RL biosynthesis genes were transformed into subcloning efficient *E. coli* DH5α competent cells (F^−^ Φ80*lac*ZΔM15 Δ(*lac*ZYA-*arg*F) U169 *rec*A1 *end*A1 *hsd*R17(r_k_^−^, m_k_^+^) *pho*A *sup*E44 *thi*-1 *gyr*A96 *rel*A1 λ^−^) (*Thermo Fisher Scientific*). A 2-μl sample of each DNA ligation was added to 50 μl of *E. coli* DH5α. DNA and cells were incubated on ice for 20 min then heat shocked at 42 °C for 30 s and returned to ice for a further 2 min. Transformation reactions were then recovered in 950 μl of SOC medium and incubated for 1 h at 37 °C. Following recovery, 100 μl of each transformation was plated onto nutrient broth (NB) agar (*Oxoid*) supplemented with 100 μg ml^−1^ ampicillin, 80 μg ml^−1^ X-Gal and 50 μM IPTG (Sigma-Aldrich) and incubated at 37 °C for 18 h (Sambrook and Russell 2001). Transformant colonies were selected for further analysis based on blue/white selection and incubated for 18 h at 37 °C in 10 ml NB supplemented with 100 μg ml^−1^ ampicillin. Plasmid DNA was recovered from each selected transformant strain using a QIAprep Spin Miniprep Kit (*Qiagen*) following the manufacturer’s instructions. The presence of insert DNA in the recovered plasmids was confirmed by a restriction enzyme digest using *Eco*RI (Sigma-Aldrich) carried out as per the manufacturer’s instructions, followed by separation on a 1% (*w*/*v*) agarose gel as previously described. Plasmid DNA with the correct size insert fragments was sequenced using the Sanger sequencing technique by *GATC Biotech* (Cologne, Germany) with the M13 forward and reverse primers.

### *Galleria mellonella* infection model

Assessment of the virulence of MCTG214(3b1) using the *G. mellonella* infection model was carried out using a protocol based on Hill et al. (2014). As a comparative positive control for these experiments, *P. aeruginosa* PAO1 (ATCC 15692) was routinely cultured at 37 °C in NB (*Oxoid*). Ten millilitres of stationary phase culture was centrifuged (10,000×*g*; 20 min) to pellet the cells, which were then washed in sterile phosphate-buffered saline (PBS). Washed cells were re-suspended to OD_600 nm_ 0.4 in PBS. The CFU count at OD_600 nm_ 0.4 was established using the Miles and Misra method and the samples diluted in sterile PBS to a concentration of 1 × 10^3^ CFU ml^−1^. *G. mellonella* larvae were purchased from *Pets at Home* (Belfast) and larvae of approx. 20 mm in length and 200 mg in weight were selected. Twenty microlitres of either bacterial sample or PBS (negative control) was injected using a 0.30 mm (30G) × 8 mm hypodermic needle (*BD*) into the last pro-leg of individual larvae (*n* = 10 per experimental group) and the larvae then incubated at 37 °C for 48 h. The larvae were observed at set time points during the course of the 48-h period and recorded as either live or dead for each individual larva at each time point. The experiment was carried out on three independent occasions and the data pooled to give and n of 30 per experimental group (Hill et al. 2014).

#### Sequence data

All sequencing data was submitted prior to publication to the GeneBank Centre (NCBI, USA), and the accession numbers were MG786779 (16S rDNA), MG805347 (*gryB*), MG805348 (*rhlA*) and MG805349 (*rhlB*).

## Results

### Isolation of and identification of strain MCTG214(3b1)

Strain MCTG214(3b1) was isolated from an enrichment of PAHs of a phytoplankton bloom field sample collected in the summer of 2009 at Sarasota Bay (USA). Selection of this strain for further study was based on preliminary screening for surface tension reduction, emulsification, gelling and foaming ability (data not shown). Taxonomic identification of MCTG214(3b1) was ascertained by sequencing and BLASTn analysis of two phylogenetic markers the 16S rRNA gene (GeneBank MG786779) and the DNA gyrase subunit B gene (*gryB*) (GeneBank MG805347) showed MCTG214(3b1) to belong to the genus *Pseudomonas*. Comparison via sequence alignment to each marker gene from the type strains of 21 separate species belonging to the genus *Pseudomonas* showed MCTG214(3b1) to be closely related to both *Pseudomonas pseudoalcaligenes* and *Pseudomonas oleovorans* based on 16S rRNA and *Pseudomonas mendocina* based on *gyrB* (Fig. S1). The new isolate, identified as *Pseudomonas* sp. MCTG214(3b1), was deposited in the Deutsche Sammlung von Mikroorganismen und Zellkulturen (DSMZ) culture collection (DSM 105446).

### Growth and surface tension reducing ability of *Pseudomonas* sp. MCTG214(3b1)

The growth of *Pseudomonas* sp. MCTG214(3b1) in ZMB was assessed at a range of temperatures, initial medium pH, medium salinity, defined carbon/nitrogen source and in the presence/absence of Zobell’s marine supplements (Fig. S2). Incubation temperature and presence/absence of Zobell’s marine supplements showed no significant effect on bacterial growth. Initial medium pH of 5.5 and 8.5 showed no significant effect on bacterial growth when compared to growth in media with an initial pH of 7.0; however, growth was significantly reduced in media with an initial pH of 4. Bacterial growth in the absence (0 g l^−1^) of sea salts was significantly reduced when compared to growth in 5–40 g l^−1^ sea salts; however, there was no significant effect on bacterial growth in medium containing between 5 and 40 g l^−1^ sea salts. Bacterial growth in medium containing urea, NaNO_3_ or NH_4_NO_3_ as a defined nitrogen source was significantly reduced compared to growth in a medium where the defined nitrogen source was a mixture of peptone and yeast extract. Finally, bacterial growth was significantly increased in the presence of 1% (*w*/*v*) glucose compared to an absence of a defined carbon source.

**Fig. 1:**
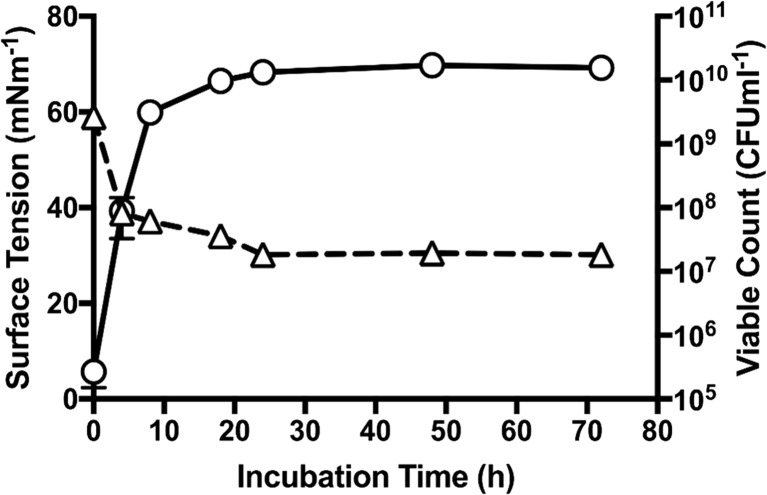
Cell-free supernatant samples obtained from *Pseudomonas* sp. MCTG214(3b1) during growth in ZMB supplemented with rapeseed oil show a reduction in surface tension during the late-exponential and stationary growth phases. Symbols: surface tension (∆) and biomass (○). Error bars represent standard deviation from the mean (*n* = 3)

Bacterial growth and the ability of *Pseudomonas* sp. MCTG214(3b1) to reduce surface tension at an air-liquid interface was assessed throughout a 72-h growth period in ZMB supplemented with 1% (*v*/*v*) rapeseed oil. When compared to samples obtained from cultures at *t* = 0 h, the surface tension was significantly reduced in samples measured from the late exponential growth phase through to stationary growth phase (Fig. 1). The effect on surface tension was most profound during the early stationary phase of growth (approx. *t* = 24 h) where mean surface tension was measured at 30.13 mN M^−1^ (± 0.20 mN M^−1^). This reduction in surface tension was observed to be relatively constant throughout the stationary growth phase (t = 24 h–*t* = 72 h).

### Chemical analysis of biosurfactant synthesis

**Fig. 2:**
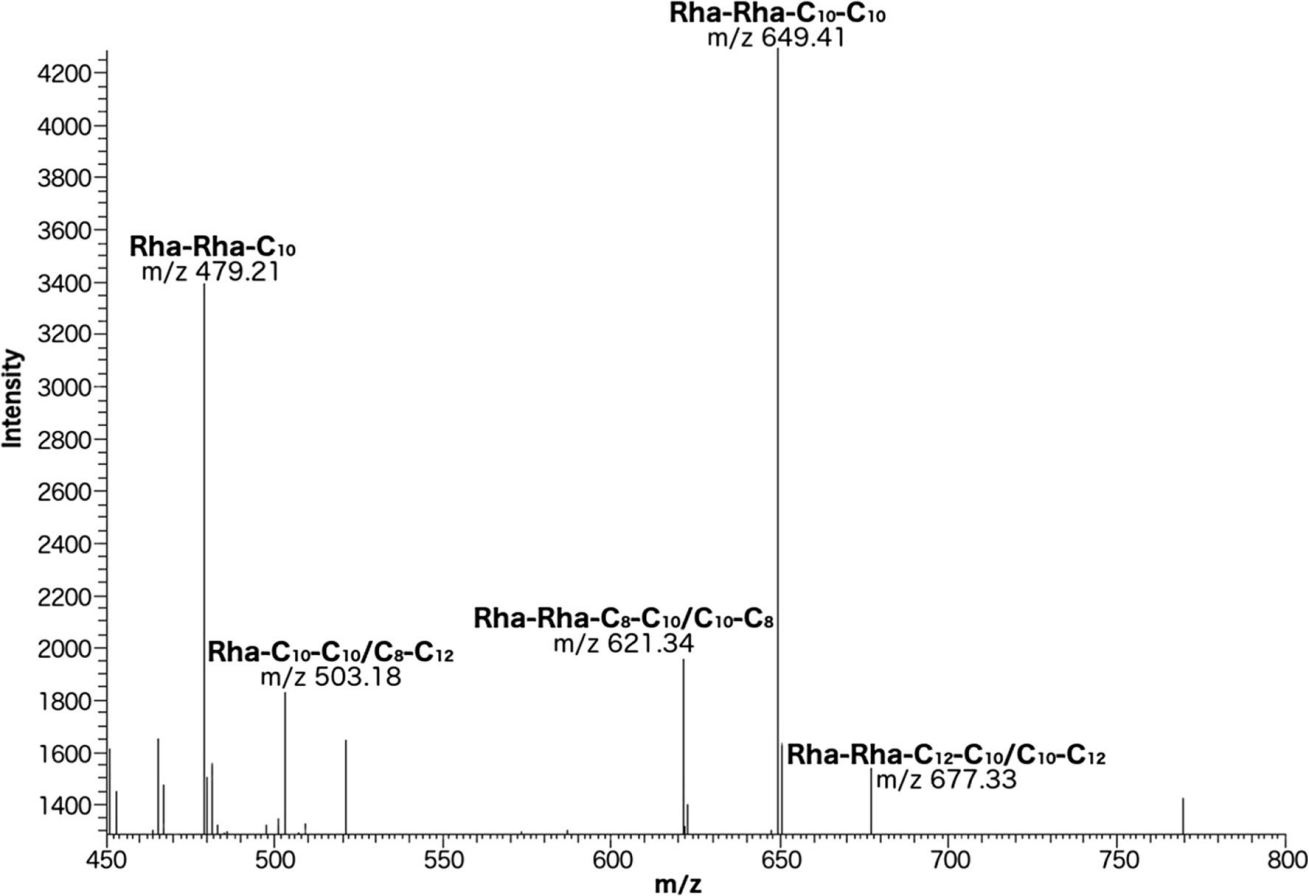
HPLC-MS profile of a SPE purified extract obtained from *Pseudomonas* sp. MCTG214(3b1) cell-free supernatant samples. The predominant peaks were identified as Rha-Rha-C_10_ and Rha-Rha-C_10_-C_10_

The ability of *Pseudomonas* sp. MCTG214(3b1) to reduce the surface tension of the culture supernatant during the stationary growth phase suggested the synthesis of a low molecular weight biosurfactant. To assess this crude liquid phase, solvent extract obtained from cell-free stationary phase culture supernatant was obtained and gravimetrically assessed. Crude extract obtained from cultures of *Pseudomonas* sp. MCTG214(3b1) showed a biosurfactant yield of 2.6 g l^−1^. Following further purification using SPE columns, purified biosurfactant yield was shown to be 0.457 g l^−1^. To elucidate the chemical structure of the biosurfactant(s) synthesised by *Pseudomonas* sp. MCTG214(3b1), the purified extract was analysed by both HPLC-MS and NMR spectroscopy. The HPLC-MS profile obtained from SPE purified extracted supernatant samples of *Pseudomonas* sp. MCTG214(3b1) showed the presence of predominant peaks corresponding to the molecular weight of five rhamnolipid congeners (Fig. 2). HPLC-MS produced a good separation of these RL congeners and the relative percentage of each congener was ascertained (Table 1). Four out of five of these RL congeners were di-rhamnolipids and accounted for a total relative abundance of 87.74%. The two predominantly abundant congeners synthesised by *Pseudomonas* sp. MCTG214(3b1) were Rha-Rha-C_10_-C_10_ (42.75%) and Rha-Rha-C_10_ (23.80%). From this analysis, no rhamnolipid congeners possessing fatty acid chains longer than 12 carbon are synthesised by *Pseudomonas* sp. MCTG214(3b1).

**Table 1:** Rhamnolipid congeners synthesised by *Pseudomonas* sp. MCTG214(3b1) and the percentage relative abundance of each congener

RT min	m/z	Compound	M_w_	Mol. Form	Relative %
13.31	479.21	Rha-Rha-C_10_	480.55	C_22_H_40_O_11_	23.80
13.36	649.41	Rha-Rha-C_10_-C_10_	650.79	C_32_H_58_O_13_	42.74
14.29	503.18	Rha-C_10_-C_10_/C_8_-C_12_	504.65	C_26_H_48_O_9_	12.26
14.54	677.33	Rha-Rha-C_10_-C_12_/C_12_-C_10_	678.84	C_34_H_62_O_13_	9.78
32.31	621.34	Rha-Rha-C_8_-C_10_/C_10_-C_8_	622.74	C_30_H_54_O_13_	11.42

**Fig. 3:**
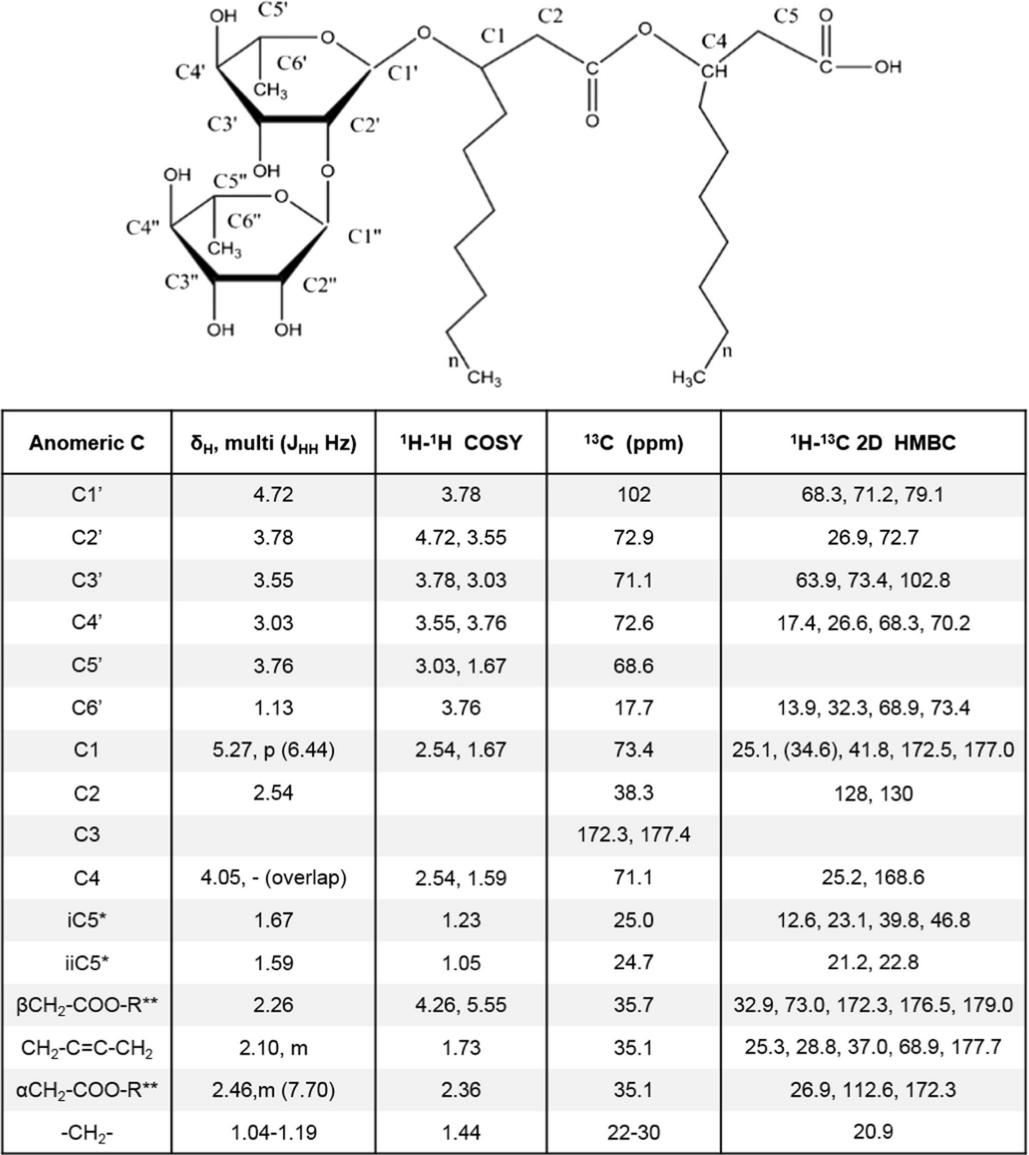
NMR resonance assignment of SPE purified supernatant extracts obtained from *Pseudomonas* sp. MCTG214(3b1). The table reports the chemical shifts of hydrogen and carbon nuclei, multiplicity of the peak and coupling constants. The diagram above the table shows the generic molecular structure of a RL congener with carbon atoms of the rhamnose unit numbered from 1′-6′ and acyl chain from 1 to 5, this numbering relates to the numbering of anomeric carbon within the table

NMR data from the ^1^H-^13^C 2D HSQC spectrum exhibited six distinct spin systems (4.60–5.50 to 92.4–102.4 ppm) indicating the presence of sugar ring moieties in the SPE-purified extracted supernatant samples of MCTG214(3b1). The presence of the carbonyl ester protons at 5.27 ppm (p, 6.44 Hz) along with the signals arising from the protons located in the sugar ring provides strong experimental evidence for the presence of glycolipid (data not shown). These data were also supported by the scalar connectivities identified between these protons in ^1^H-^1^H 2D COSY and ^1^H-^13^C 2D HMBC spectra. In the ^1^H-^1^H 2D COSY spectrum, through-bond correlations between anomeric protons and the adjacent protons of the sugar unit were observed. A strong correlation was also observed between a methyl group at 1.13 ppm and a proton at 3.76 ppm placing the methyl group at position C5 in a pentose ring indicating the existence of a rhamnose ring (Fig. 3). The proposed structure was fully supported by additional, long-range through-bond, correlations from ^1^H-^13^C 2D HMBC spectrum indicating that the sample obtained from the extraction of MCTG214 contained a rhamnolipid profile (Fig. 3). These data in combination with HPLC-MS data clearly support the synthesis of rhamnolipid by *Pseudomonas* sp. MCTG214(3b1).

### Screening for RL synthesis genes

**Fig. 4:**
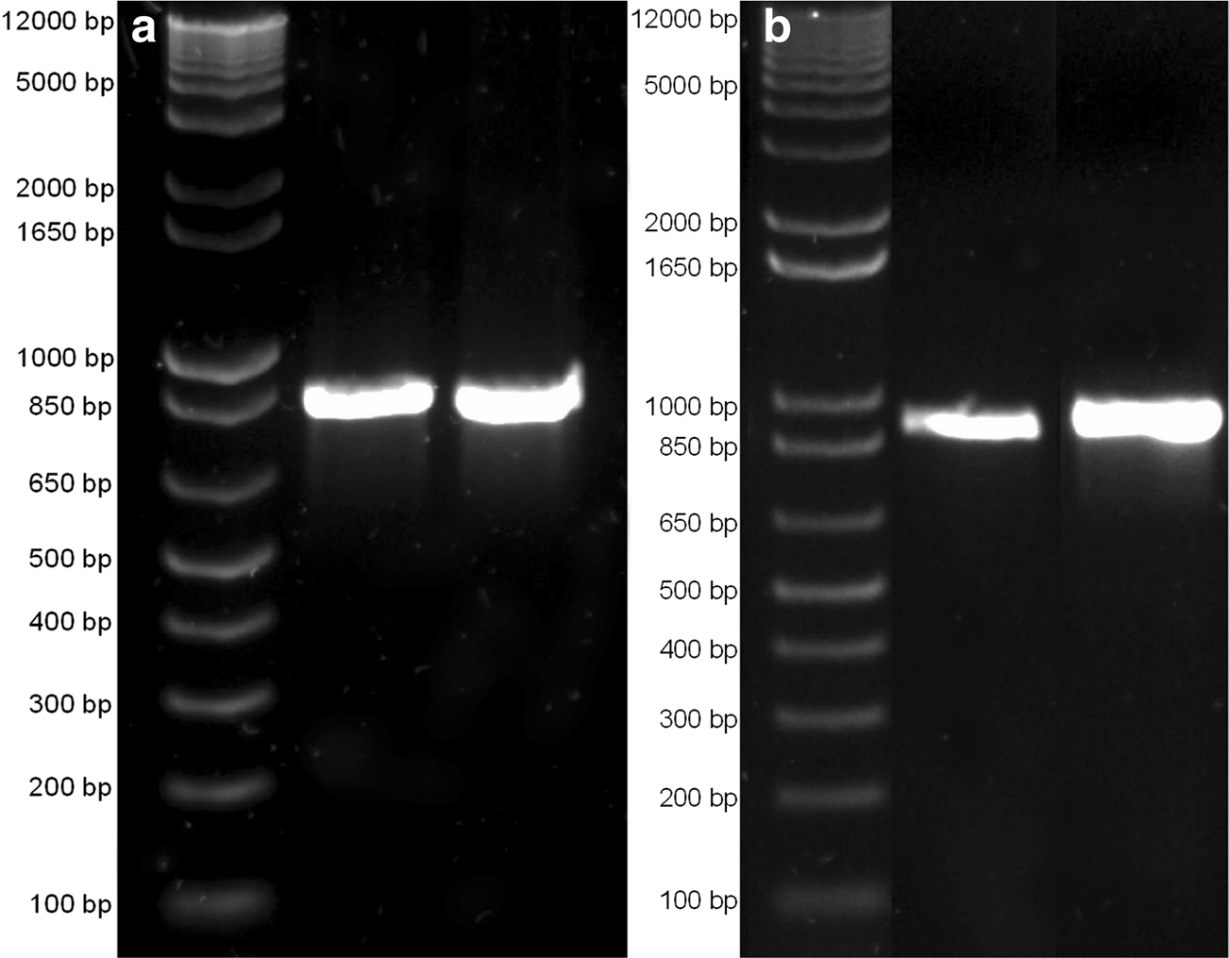
DNA fragments resulting from PCR amplification of rhamnolipid synthesis genes *rhlA* (**a**) and *rhlB* (**b**). PCR products were separated by molecular weight on a 1.5% (*w*/*v*) agarose gel, imaged under UV light using SybrSafe DNA strains (*Thermo Fisher Scientific*). Samples from left to right on each gel; 1 kb Plus DNA marker (*Thermo Fisher Scientific*), amplification product from *P. aeruginosa* PAO1, amplification product from *Pseudomonas* sp. MCTG214(3b1)

In *P. aeruginosa*, rhamnolipids are synthesised by enzymes expressed from the genes *rhlA*, *rhlB* and *rhlC*. Using primers designed to bind within the coding region of these three *P. aeruginosa* genes, the presence of *rhlA*, *rhlB* and *rhlC* in genomic DNA extracted from *Pseudomonas* sp. MCTG214(3b1) was screened by PCR. PCR screening using *rhlA* and *rhlB* primers amplified products of the same molecular weight (approx. 850 and 900 bp, respectively), when chromosomal DNA extracted from both *Pseudomonas* sp. MCTG214(3b1) and *P. aeruginosa* PAO1 DNA was provided has a template (Fig. 4). PCR screening using primers specific for *rhlC* amplified no product in *Pseudomonas* sp. MCTG214(3b1) matching the molecular weight of that amplified in *P. aeruginosa* PAO1. Additional screening using primers designed from the coding region of the *B. thailandensis rhlC* homologue also failed to amplify any product in *Pseudomonas* sp. MCTG214(3b1). The putative *rhlA* and *rhlB* DNA amplified from *Pseudomonas* sp. MCTG214(3b1) chromosomal DNA was subsequently cloned into pGEM-T Easy and sequenced from the purified plasmid (*rhlA*–GeneBank MG805348 and *rhlB*–GeneBank MG805349). BLASTn sequence analysis showed that the DNA amplified from *Pseudomonas* sp. MCTG214(3b1) matched *rhlA* and *rhlB* from multiple strains of *P. aeruginosa* with a sequence homology averaging 99%. Further analysis showed that the translated *rhlA* and *rhlB* sequences of MCTG214(3b1) possessed an average 96% amino acid sequence identity to RhlA sequences obtained from multiple *P. aeruginosa* strains and 98% amino acid sequence identity to RhlB sequences obtained from the same multiple *P. aeruginosa* strains.

### Assessment of virulence using the *Galleria mellonella* infection model

**Fig. 5:**
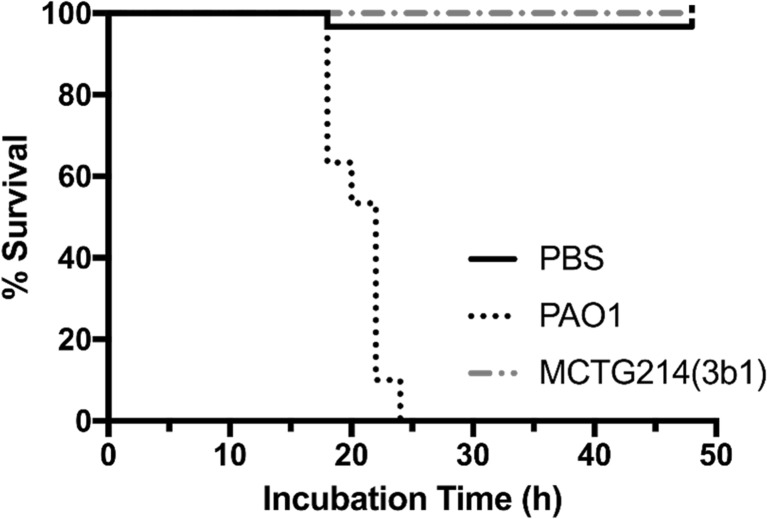
Kaplan-Meier plot showing percentage survival of *Galleria mellonella* larvae after inoculation with either *Pseudomonas* sp. MCTG214(3b1) or *P. aeruginosa* PAO1. No significant mortality was observed after infection with *Pseudomonas* sp. MCTG214(3b1) within a 48-h incubation as opposed to infection with *P. aeruginosa* PAO1 where 100% mortality was observed within 24-h incubation. No significant mortality was observed in larvae inoculated with the carrier control buffer (PBS). *n* = 30 (pooled from 3× duplicate experiments)

The potential virulence of *Pseudomonas* sp. MCTG214(3b1) was assessed and compared to that of the RL producing opportunistic pathogen *P. aeruginosa*. *P. aeruginosa* PAO1 was shown to kill 100% of the infected larvae 24 h post-inoculation with as little as 20 CFU (Fig. 5). When inoculated with an equal CFU count of *Pseudomonas* sp. MCTG214(3b1) or with sterile PBS, the larvae showed 100% survival 48 h post-inoculation, (Fig. 5).

## Discussion

Biosurfactants such as RLs are increasingly viewed by industry as attractive replacements for artificially synthesised surfactant compounds with applications in the home care, food, pharmaceutical and petrochemical sectors (Banat et al. 2010). Whilst the best-characterised bacterium to synthesise RLs is *P. aeruginosa*, large-scale culture of this species is problematic due to this organism’s status as a biological safety level 2 pathogen (Soberón-Chávez et al. 2005; Abdel-Mawgoud et al. 2010). Bacteria are thought to synthesise surface-active compounds such as RLs, in part to help solubilise poorly soluble substrates such as hydrocarbons and hence enhance their bioavailability and subsequent metabolism (Abdel-Mawgoud et al. 2010). Several studies have reported the isolation of hydrocarbon-degrading bacteria from laboratory cultures of phytoplankton across the three main lineages (diatoms, dinoflagellates and coccolithophores) (Green et al. 2004; Green et al. 2006; Gutierrez et al. 2012a; Gutierrez et al. 2012b; Gutierrez et al. 2013; Gutierrez et al. 2014; Mishamandani et al. 2016). Some were identified through sequencing surveys in field samples of phytoplankton (Gutierrez et al. 2011; Thompson et al. 2017). The occurrence of hydrocarbon-degrading bacteria with phytoplankton may be more frequent and important in the global ocean environment than previously perceived, including as a source for discovering novel types of biosurfactants such as RLs. Whilst there is a plethora of previous studies reporting RL production in bacteria other than *P. aeruginosa*, many of them either fail to prove RL synthesis utilising robust chemical analysis techniques or they do not provide accurate identification of the strain investigated. Irorere et al. (2017) recently published a number of criteria for how to confirm bacterial biosurfactant production, such as, for example using HPLC-MS for the analysis of supernatant extracts, detection of biosurfactant synthesis genes using molecular biology tools and phylogenetic identification of the strains of interest (Irorere et al. 2017). Here, we employ these criteria to confirm the production of RLs by the new isolate *Pseudomonas* sp. MCTG214(3b1).

The sequencing of the two phylogenetic markers, 16S rDNA and DNA gyrase B (*gyrB*) produced conflicting results since using the former identified the strain as *Pseudomonas pseudoalcaligenes*, whereas with *gyrB*, the strain was identified as *Pseudomonas mendocina*. Based on DNA-DNA relatedness and molecular profiling, *Pseudomonas pseudoalcaligenes* has now been re-classified as *Pseudomonas oleovorans* (Saha et al. 2010). Both *P. oleovorans and P. mendocina* have been previously isolated from varying environmental niches, including the marine environment (Xu et al. 2006; Shi et al. 2009; Mangwani et al. 2014). A study investigating taxonomic relatedness of *Pseudomonas* species using 112 draft *Pseudomonas* genomes and 141 well-characterised *Pseudomonas* strains showed 16 subgroups to be present within the genus (Gomila et al. 2015). *P. oleovorans* and *P. mendocina* belong to the same clade within the genus, which is separate from the clade containing *P. aeruginosa* (Gomila et al. 2015). There is limited evidence that these two species can cause infection; however, these cases appear to be confined to persons with compromised immune systems (Johansen et al. 2001; Faccone et al. 2014; Gautam et al. 2015). To ascertain that *Pseudomonas* sp. MCTG214(3b1) is less pathogenic than *P. aeruginosa*, this study utilised the *G. mellonella* model. *G. mellonella* larvae possess a basic innate immune system analogous to a neutrophil response in humans, and it has been used in multiple studies investigating virulence in a number of bacterial species (Mukherjee et al. 2011; Tsai et al. 2016). Larvae inoculated with *Pseudomonas* sp. MCTG214(3b1) showed no pathogenic effect on the *G. mellonella* after 48-h incubation at 37 °C compared to those inoculated with the same CFU of *P. aeruginosa* PAO1 where 100% mortality was observed within 24-h incubation. In addition to a lack of any observable pathogenicity to *G. mellonella*, strains of both *P. oleovorans* and *P. mendocina* deposited in various culture collections, including the ATCC, are reported to be biological safety category 1.

Analysis of cell-free supernatant samples obtained from stationary phase cultures of *Pseudomonas* sp. MCTG214(3b1) showed significant reduction in surface tension. This profile of surface tension reduction is similar to that previously observed and attributed to RL production in *P. aeruginosa* (Ismail et al. 2015). To conclusively link this observation of surface tension reduction in *Pseudomonas* sp. MCTG214(3b1) cultures with the production of biosurfactant compounds, the qualitative analytical techniques, HPLC-MS and NMR spectroscopy, were employed. HPLC-MS spectra obtained from the SPE-purified supernatant extracts showed peaks indicative of five separate RL congeners, a significant majority which were shown to possess a molecular structure that included two rhamnose moieties, i.e. di-rhamnolipids. The synthesis of RL congeners by *Pseudomonas* sp. MCTG214(3b1) identified by HPLC-MS was supported by data from NMR spectroscopy analysis which elucidated ^1^H and ^13^C resonances indicative of glycolipid molecules containing rhamnose moieties. The di-rhamnolipid congeners synthesised by this strain totalled a relative abundance of 87.74%. Although a strong prevalence towards di-rhamnolipid synthesis in bacteria such as *P. aeruginosa* and *B. thailandensis* is commonly reported, this level of di-rhamnolipid abundance appears to be significantly higher than in other wild-type strains (Rudden et al. 2015; Funston et al. 2016). The virtually exclusive synthesis of di-rhamnolipids by *Pseudomonas* sp. MCTG214(3b1) is advantageous for biotechnological application due to no genetic manipulation of the strain being required to select for these congeners. The β-hydroxy fatty acid moieties incorporated into RL congeners synthesised by *Pseudomonas* sp. MCTG214(3b1) varied between 8 and 12 carbons in length, similar to those shown to be incorporated into RLs synthesised by *P. aeruginosa* (Rudden et al. 2015). This differs from those incorporated by *B. thailandensis* which shows a preference towards fatty acids of longer (12–14 carbons) chain length (Funston et al. 2016). Indeed, the RL congener shown to be most prevalently synthesised by *Pseudomonas* sp. MCTG214(3b1), Rha-Rha-C_10_-C_10_, is identical to that which is most prevalently synthesised by *P. aeruginosa* (Rudden et al. 2015). Although the profile of di-rhamnolipids synthesised by *Pseudomonas* sp. MCTG214(3b1) is highly similar to *P. aeruginosa*, there are differences such as the predominant synthesis of congener Rha-Rha-C_10_, which appears to be unique to this strain.

Using RL-specific gene primers for PCR, this study identified *Pseudomonas* sp. MCTG214(3b1) to possess homologues to the rhamnolipid synthesis genes *rhlA* and *rhlB*. The combination of both enzymes encoded by these genes is responsible for the rhamnosyltransferase that synthesises mono-rhamnolipids. RhlA forms the HAA precursor molecules and RhlB adds rhamnose sugar moieties to form the completed RL (Ochsner et al. 1994; Rahim et al. 2001; Deziel et al. 2003). The presence of these two genes indicates *Pseudomonas* sp. MCTG214(3b1) is genetically capable of RL synthesis. The DNA sequence of *rhlA* and *rhlB* amplified from *Pseudomonas* sp. MCTG214(3b1) possessed over 98% homology to *rhlA* and *rhlB* in *P. aeruginosa*. The high level of sequence homology to *P. aeruginosa* contrasts to the observation of Funston et al. (2016) who showed *rhlA*/*B* homologues present in *B. thailandensis* that were below 50% homology to those present in *P. aeruginosa* (Funston et al. 2016). The high level of sequence homology to *P. aeruginosa* may indicate evolution of the rhamnosyltransferase gene operon within the *Pseudomonas* genus from a joint ancestor or horizontal gene transfer within the genus. The high level of *rhlA* sequence homology between *P. aeruginosa* and *Pseudomonas* sp. MCTG214(3b1) may cause the expression of enzymes of similar high amino acid sequence identity and therefore corresponding substrate prevalence. This could account for the comparable synthesis of short chain RLs to those synthesised by *P. aeruginosa* and contrast to those synthesised by *B. thailandensis* where sequence identity is low.

The mechanism for di-rhamnolipid synthesis in both *P. aeruginosa* and *B. thailandensis* is well established with the process involving the addition of a second rhamnose sugar to the mono-rhamnolipid already generated by the actions of RhlA/B (Ochsner et al. 1994; Deziel et al. 2003; Zhu and Rock 2008; Dubeau et al. 2009). The addition of the second rhamnose is catalysed by a second rhamnosyltransferase enzyme RhlC (Rahim et al. 2001). In *P. aeruginosa*, this second rhamnosyltransferase is expressed from the gene *rhlC* located approximately 1 Mb downstream on the genome from the *rhlA*/*B* genes (Stover et al. 2000). The *B. thailandensis rhlC* homologue is found within the same operon as the *rhlA*/*B* homologues (Dubeau et al. 2009). As with the *rhlA*/*B* genes, *rhlC* found in *P. aeruginosa* shares a low sequence homology to its functional homologue in *B. thailandensis* (Dubeau et al. 2009; Funston et al. 2016). HPLC-MS analysis of *Pseudomonas* sp. MCTG214(3b1) products clearly identified di-rhamnolipid congeners, and it was expected that this strain would also possess an *rhlC* homologue with high sequence homology to *P. aeruginosa*. When chromosomal DNA from *Pseudomonas* sp. MCTG214(3b1) was probed using primers designed for identifying this gene in *P. aeruginosa*, no amplified product was identified. Further screening using primers designed for *B. thailandensis* also yielded negative results.

The clear production of di-rhamnolipid by *Pseudomonas* sp. MCTG214(3b1) and lack of *rhlC* presents an anomaly. If the *rhlA*/*B* genes in *Pseudomonas* sp. MCTG214(3b1) were acquired via horizontal gene transfer from *P. aeruginosa*, it is feasible that *rhlC* would not have been acquired in the same manner due to the sizeable gap between the *rhlA*/*B* genes and *rhlC* in *P. aeruginosa* (Stover et al. 2000; Deziel et al. 2003). We therefore postulate that di-rhamnolipid synthesis observed in *Pseudomonas* sp. MCTG214(3b1) is catalysed by a novel second rhamnosyltransferase. We also posit that this would account for the subtle differences in RL congener profile in *Pseudomonas* sp. MCTG214(3b1) compared to that of *P. aeruginosa*, and importantly the observation of nearly total conversion of mono-rhamnolipid to di-rhamnolipid. Based on the RL profile of *Pseudomonas* sp. MCTG214(3b1), this novel RhlC is likely to have a higher substrate affinity for mono-rhamnolipid than RhlC from either *P. aeruginosa* or *B. thailandensis* where the reported relative abundances of di-rhamnolipid are 62.85 and 79.33%, respectively (Rudden et al. 2015; Funston et al. 2016). Differences in gene expression profile to account for the near total conversion of mono-rhamnolipid by *Pseudomonas* sp. MCTG214(3b1) can be discounted since the surface tension reduction profiles of both *Pseudomonas* sp. MCTG214(3b1) and *P. aeruginosa* are highly similar. The identification and characterisation of this putative *rhlC* gene responsible for the near exclusive di-rhamnolipid synthesis and the analysis of the expression of *rhlA*/*B* homologues identified in this study will be the focus of future work. Future work will also include an investigation of regulatory pathways that govern RL synthesis in *Pseudomonas* sp. MCTG214(3b1) which, as in both *P. aeruginosa* and *B. thailandensis*, may be modulated through AHL-mediated quorum sensing systems (Pearson et al. 1997; Funston et al. 2016).

This study has extended the number of species producing RL within the *Pseudomonas* genus to include a previously un-investigated marine strain MCTG214(3b1). Confirmed identification of RL production in a bacterial strain isolated from phytoplankton bloom highlights marine phytoplankton as an untapped resource in the ocean for the discovery of novel types of biosurfactants. Additionally, the discovery that this strain in its wild-type form appears to synthesise a significantly high proportion of di-rhamnolipid may have important biotechnological application that can be further investigated.

## Electronic supplementary material

ESM 1 (PDF 359 kb)
