# Supplementary material for: Identification and characterisation of short chain rhamnolipid production in a previously uninvestigated, non-pathogenic marine pseudomonad
Source: Appl Microbiol Biotechnol. 2018 Jul 10;102(19):8537–49. doi: 10.1007/s00253-018-9202-3 (PMC6153872; doi:10.1007/s00253-018-9202-3)
Supplement: Supplementary file 1 — (PDF 359 kb) [file 253_2018_9202_MOESM1_ESM.pdf]

APPLIED MICROBIOLOGY AND BIOTECHNOLOGY

Supplementary material for:

Identification and Characterisation of Short Chain Rhamnolipid Production in a Previously Uninvestigated, Non-Pathogenic Marine Pseudomonad.

Twigg, M.S.[1*], Tripathi, L.[1], Zompra, A.[3], Salek, K.[2], Irorere, V.U.[1], Gutierrez, T.[2], Spyroulias, G.A.[3], Marchant, R.[1] & Banat, I.M.[1]

***Corresponding Author - Matthew S. Twigg**

m.twigg@ulster.ac.uk Tel: +44(0)2870123097

[1]School of Biomedical Sciences, Ulster University, Coleraine, BT52 1SA, Northern Ireland, UK.

[2]Institute of Mechanical, Process & Energy Engineering, School of Engineering & Physical Sciences, Heriot-Watt University, Edinburgh, EH14 4AS, UK.

[3]Department of Pharmacy, University of Patras, 26504, Patras, Greece.

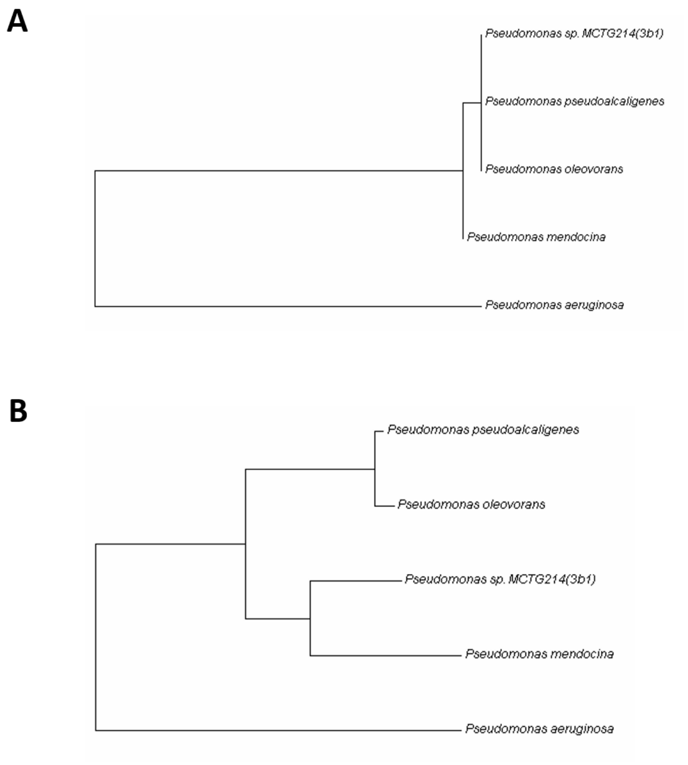
A
Pseudomonas sp. MCTG214(3b1)
Pseudomonas pseudoalcaligenes
Pseudomonas oleovorans
Pseudomonas mendocina
Pseudomonas aeruginosa
B
Pseudomonas pseudoalcaligenes
Pseudomonas oleovorans
Pseudomonas sp. MCTG214(3b1)
Pseudomonas mendocina
Pseudomonas aeruginosa

Figure S1.

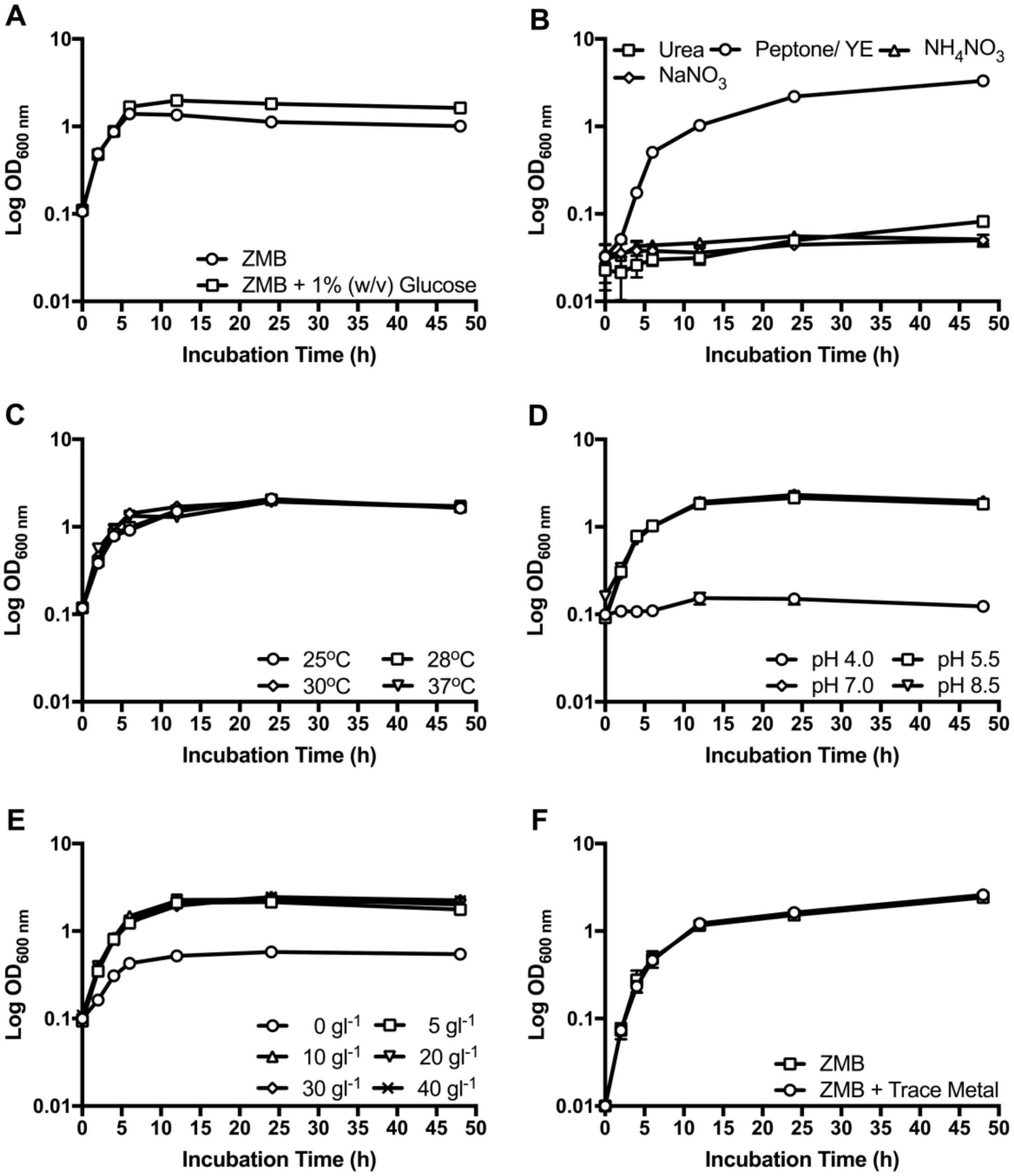

Figure S2.

Supplementary Figure Legends

Figure S1. Phylogenetic trees resulting from sequence alignments of *Pseudomonas sp.* MCTG214(3b1) 16S rDNA (**A**) and *gyrB* (**B**) against the sequences *Pseudomonas* type species shown via BLAST to have high levels of sequences identity. The comparative sequences were obtained from Genbank. The tree topology is based on

neighbour-joining and bootstrap analysis was performed with 1000 replications. Evolutionary analyses were conducted in MEGA7.

Figure S2**.** Growth of *Pseudomonas sp.* MCTG214(3b1) in different culture conditions. The growth of *Pseudomonas sp.* MCTG214(3b1) cultured in ZMB was assessed over a 48 hour growth cycle whilst exposing the cells to varying physical and medium conditions **A.** absence/ presence of a defined carbon source (glucose); **B.** in the presence of defined nitrogen source (Urea, peptone/ YE, NH_4NO_3 and $NaNO_3$); **C.** temperature range (25°C – 37°C); **D.** initial media pH (pH 4.0 – 8.5); **E.** defined concentration of sea salt within the media (0 gl^{-1} – 40 gl^{-1} sea salt); **F.** absence/ presence of trace metal supplements. (n = 3, error bars represent standard deviation from the mean).

Table S1

Primer	Description	DNA Seq. (5’-3’)	T_m	Ref
9bfm	Forward primer used for the amplification and sequencing of 16S rDNA corresponding to bp 9 of the *E. coli* 16S rDNA gene. Amplicon size when used with primer 1512uR is approx. 1500 bp.	GAGTTTGATYHTGGCTCAG	52°C	Mühling et al. 2008
1512uR	Reverse primer used for the amplification and sequencing of 16S rDNA corresponding to bp 1512 of the *E. coli* 16S rDNA gene. Amplicon size when used with primer 9bfm is approx. 1500 bp.	ACGGHTACCTTGTTACGACTT	53 ^{o}C	Mühling et al. 2008
536F	Internal primer used for the sequencing of 16S rDNA corresponding to bp position 536 of the *E. coli* 16S rDNA gene.	GTGCCAGCMGCCGCGGTAATWC	N/A	Morales *et al.*, 2009
907R	Internal primer used for the sequencing of 16S rDNA corresponding to bp position 907 of the *E. coli* 16S rDNA gene.	CCGTCAATTCMTTTGAGTTT	N/A	Morales *et al.*, 2009
UP-1	Degenerate forward primer designed for the amplification of *gyrB* DNA. Amplicon size when used with primer UP-2r is approx. 1200 bp.	GAAGTCATCATGACCGTTCTGCAYGC NGGNAARTTYGA	57°C	Yamamoto and Harayama 1995
UP-2r	Degenerate reverse primer designed for the amplification of *gyrB* DNA. Amplicon size when used with primer UP-1 is approx. 1200 bp.	AGCAGGGTACGGATGTGCGAGCCRTC NACRTCNGCRTCNGTCAT	57°C	Yamamoto and Harayama 1995
rhlA-F	Forward primer for amplification of *rhlA,* designed from multiple *rhlA* sequences from *P. aeruginosa.* Amplicon size when used with primer *rhlA*-R is approx. 850 bp.	GCATTCGCCCCTGGACTGA	52°C	This study
rhlA-R	Reverse primer for amplification of *rhlA,* designed from multiple *rhlA* sequences from *P. aeruginosa.* Amplicon size when used with primer *rhlA*-F is approx. 850 bp.	ATGTGCTGATGGTTGCTGGCTTT	52°C	This study

rhlB-F	Forward primer for amplification of *rhlB,* designed from multiple *rhlB* sequences from *P. aeruginosa.* Amplicon size when used with primer *rhlB*-R is approx. 900 bp.	CACGCCATCCTCATCGCC	50°C	This study
rhlB-R	Reverse primer for amplification of *rhlB,* designed from multiple *rhlB* sequences from *P. aeruginosa.* Amplicon size when used with primer *rhlB*-F is approx. 900 bp.	GGTCAGTTCGTCGCTCAGC	50°C	This study
rhlC-F	Forward primer for amplification of *rhlC,* designed from multiple *rhlC* sequences from *P. aeruginosa.* Amplicon size when used with primer *rhlC*-R is approx. 800 bp.	AACTGGCGGCGGCGTTTCC	51°C	This study
rhlC-R	Reverse primer for amplification of *rhlC,* designed from multiple *rhlC* sequences from *P. aeruginosa.* Amplicon size when used with primer *rhlC*-F is approx. 800 bp.	AGTCCTGGTCGAGCAGCAGCA	51°C	This study

Supplementary Table Headings

Table S1. Table listing the primers used in this study for both PCR and DNA sequencing.
